# Development of a bent Laue beam-expanding double-crystal monochromator for biomedical X-ray imaging

**DOI:** 10.1107/S1600577514003014

**Published:** 2014-03-13

**Authors:** Mercedes Martinson, Nazanin Samadi, George Belev, Bassey Bassey, Rob Lewis, Gurpreet Aulakh, Dean Chapman

**Affiliations:** a Physics and Engineering Physics, University of Saskatchewan, 116 Science Place, Room 163, Saskatoon, Saskatchewan, Canada S7N 5E2; b Biomedical Engineering, University of Saskatchewan, 107 Wiggins Road, Saskatoon, Saskatchewan, Canada S7N 5E5; c Biomedical Imaging and Therapy Beamlines, Canadian Light Source, 44 Innovation Boulevard, Saskatoon, Saskatchewan, Canada S7N 2V3; d Medical Imaging, University of Saskatchewan, 107 Wiggins Road, Saskatoon, Saskatchewan, Canada S7N 5E5; e Department of Medical Imaging and Radiation Sciences, Monash University, Clayton, Victoria 3800, Australia; f Anatomy and Cell Biology, University of Saskatchewan, 107 Wiggins Road, Saskatoon, Saskatchewan, Canada S7N 5E5

**Keywords:** beam expander, bent Laue diffraction, double-crystal monochromator, biomedical imaging, dynamic imaging

## Abstract

The Biomedical Imaging and Therapy (BMIT) beamline at the Canadian Light Source has produced some excellent biological imaging data. However, the disadvantage of a small vertical beam limits its usability in some applications. Micro-computed tomography (micro-CT) imaging requires multiple scans to produce a full projection, and certain dynamic imaging experiments are not possible. A larger vertical beam is desirable. It was cost-prohibitive to build a longer beamline that would have produced a large vertical beam. Instead, it was proposed to develop a beam expander that would create a beam appearing to originate at a source much farther away. This was accomplished using a bent Laue double-crystal monochromator in a non-dispersive divergent geometry. The design and implementation of this beam expander is presented along with results from the micro-CT and dynamic imaging tests conducted with this beam. Flux (photons per unit area per unit time) has been measured and found to be comparable with the existing flat Bragg double-crystal monochromator in use at BMIT. This increase in overall photon count is due to the enhanced bandwidth of the bent Laue configuration. Whilst the expanded beam quality is suitable for dynamic imaging and micro-CT, further work is required to improve its phase and coherence properties.

## Abstract 2

A bent Laue beam-expanding double-crystal monochromator was developed and tested at the Biomedical Imaging and Therapy beamline at the Canadian Light Source. The expander will reduce scanning time for micro-computed tomography and allow dynamic imaging that has not previously been possible at this beamline.

## 1. Introduction   

Biomedical X-ray imaging using synchrotron light sources has been well established (Suortti & Thomlinson, 2003 ▶; Lewis, 2004 ▶; Thomlinson *et al.*, 2005 ▶; Liu *et al.*, 2013 ▶; Suortti *et al.*, 2013 ▶; Coan *et al.*, 2013 ▶; Bravin *et al.*, 2013 ▶). Biomedical beamlines are in use around the world for a variety of imaging techniques including in-line phase contrast and micro-computed tomography (micro-CT). At the Canadian Light Source in Saskatoon, two biomedical beamlines have been commissioned: BMIT-BM uses a bend magnet and BMIT-ID uses a wiggler insertion device. While both of these beamlines offer high flux, they suffer the drawback of small beam heights. BMIT-BM produces a maximum beam height of approximately 7 mm at a 23 m source-to-sample distance, and BMIT-ID produces a maximum beam height of 11 mm at a 55 m source-to-sample distance. As a result, most samples must be scanned vertically through the beam to image the entire region of interest.

Vertical scanning poses severe limitations in two major areas. CT scans must be made in small vertical sections, imaging roughly 5 mm per rotation. Consecutive sections require enough overlap to reliably stitch the projections together, so regions of the subject are imaged repeatedly. Not only is this time-consuming, but it increases the delivered dose, which is problematic for live animal studies. In addition to the longer scan times, these vertical sections must then be stitched together during processing, which increases both processing time and likelihood of error.

The second, and even more important, limitation is with dynamic imaging (Lewis *et al.*, 2005 ▶). Many important physiological processes can only be understood by capturing movies of live systems. Examples include coronary angiography and functional lung imaging (Hyodo *et al.*, 1998 ▶; Hooper *et al.*, 2009 ▶; Porra *et al.*, 2011 ▶; Schültke *et al.*, 2011 ▶; Astolfo *et al.*, 2013 ▶). Scanning subjects through the beam makes it impossible to capture the entire process in one shot which represents a major limitation of the beamline for cutting-edge studies into physiological processes.

## 2. Design and implementation   

A bent Laue double-crystal monochromator was chosen to implement the expander as it allows full tunability of the energy from 20 to 100 keV. When a crystal wafer is cylindrically bent with the concave side facing the source, the diffracted beam will diverge with a virtual focus on the incident side of the crystal. Two such crystals placed in a non-dispersive divergent geometry (Suortti & Schulze, 1995 ▶) produce a beam with a vertical height proportional to the distance between the second crystal and the virtual focal point of the first crystal. The bending radius of the second crystal must be such that its focal point is the same as that of the first crystal in order to allow maximum reflection from the planes in the second crystal. The crystals are deemed to ‘match’ when their centres are parallel (same Bragg angle) and their focal points are at the same location.

Unlike optical lenses, the focal point of a crystal is a function not only of the bending radius but also of the asymmetry and Bragg angles (χ and θ_B_, respectively). In the set-up used for these experiments, the first crystal was in an ‘up-bounce’/positive-sign geometry and the second crystal was in a ‘down-bounce’/negative-sign geometry (Erola *et al.*, 1990 ▶). The relationships between focal points, 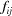, and bending radii, 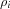, are given below. The indices denote the first (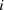 = 1) or second (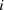 = 2) crystal, and the incident (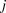 = 1) or diffracted (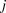 = 2) beam,
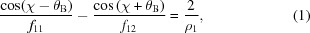


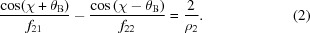
The expansion factor 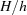 is determined by the ratio of bending radii and the crystal–crystal spacing. Suppose that an expansion of *m* times is desired. If the distance 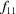 from the source to the first crystal is known and the bending radii are set such that 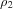 = 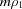 and the crystal–crystal spacing is set such that 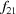 = 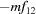, then it follows from equations (1) and (2) that 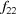 = 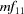. Since this double-crystal geometry preserves the divergence of the beam and beam height is proportional to vertical divergence and distance from the source, it follows that, if the spacing between the crystals is much smaller than the distance to the source, then *m* is equal to the expansion factor.

Whilst a smaller bending radius produces a larger expansion over a shorter distance, elasticity limitations of crystal wafers place lower bounds on their bending radii. Based on a rule of thumb that the minimum ‘safe’ bending radius is equal to 1000× the thickness of the crystal, it was decided that the ∼0.5 mm-thick crystals could be bent no more than ρ = 50 cm. For the preliminary attempt, the following parameters were chosen. Bending radius of first crystal: ρ_1_ = 1 m (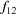 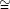 −0.5 m); bending radius of second crystal: ρ_2_ = 3 m (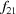 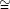 1.5 m); distance between crystals: Δ*f* = 1 m.

**Figure 1:**
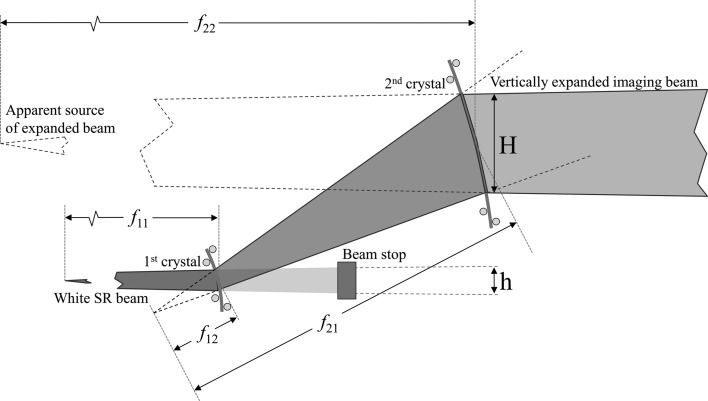
Schematic of the crystal geometry and orientation, ray-tracing diagrams and focal lengths.

The cylindrical bend was set optically and implemented using a four-bar bender (Fig. 1 ▶). This system is flexible, as virtually any bending radius can be achieved. Once the bend radius was set, the bending frames were placed on crossed goniometer stages to manipulate the Bragg angles and tilts if necessary. The appropriate reflections were found by comparing the reflection pattern produced on a fluorescent screen to stereographic projection maps. The first crystal was set to the appropriate Bragg angle for the chosen reflection and energy. The second crystal was then placed in the diffracted beam at the distance calculated from the chosen bending radii and Bragg angle. After opening the beamline slits to full size, the second crystal was aligned with the first crystal to optimize the intensity and uniformity of the beam. If necessary, the distance between crystals was adjusted in order to improve beam uniformity. For measuring beam expansion, the beamline slits were used to aperture the beam in the region with the best quality. For imaging, the beamline slits were left at full size.

A preliminary experiment was performed using (1,1,1) silicon crystal wafers with a (1,1,1)-type reflection such that χ = 19.47°. This reflection was selected due to its broad Darwin width and resulting high intensity. The Bragg angle was determined using the *K*-edge absorption of iodine as an energy calibration standard. The beam size and shape were imaged on burn paper at three locations: the incident white beam coming into the hutch, the beam diffracted by the second crystal (termed the ‘diffracted beam’), and the beam transmitted through the second crystal (the ‘transmitted beam’). Expansion was calculated as the ratio between the diffracted beam and the incident beam.

Intermediate attempts of 3×, 5× and 7× expansion were made with Si (5,1,1) crystal wafers with (2,2,0)-type reflections such that χ = 15.79°. Simple imaging tests were conducted to evaluate absorption, phase and edge features. Because the beam intensity had been more uniform during the preliminary experiment, the (1,1,1) wafers were put back in place for the high-resolution micro-CT and dynamic imaging tests.

The flux was measured using a (1,1,1)-type reflection at an energy of 20.0 keV, as confirmed by the absorption *K*-edge using a molybdenum filter. An ion chamber was placed in the expanded beam with lead shields preventing the beam from hitting the electrodes. An image of the beam through the ion chamber was captured using a 200 µm pixel size flat-panel detector (Hamamatsu C9252DK-14), allowing the exposure area to be measured precisely.

## 3. Results   

Using a (1,1,1) silicon crystal wafer with (1,1,1)-type reflections placed in matching bent Laue non-dispersive divergent geometry, the beam was expanded vertically to a maximum height seven times larger than the incident beam. The Si (5,1,1) wafers with (0,2,2)-type reflections reached a maximum expansion of 7.7×. A summary of expansion results is provided in Table 1 ▶. The target of 10× has not yet been reached and will likely require a new bending and alignment apparatus to achieve.

**Figure 2:**
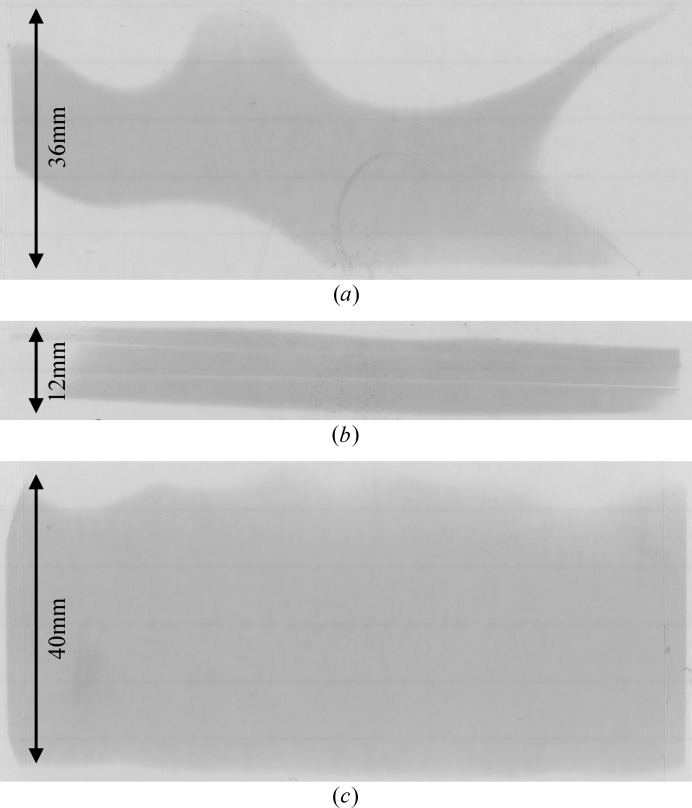
Burn paper images showing beam quality. (*a*) Extreme example of non-uniform intensity. (*b*) Beam ‘glitches’. (*c*) Large (∼40 mm) beam with uniform intensity.

The beam quality was evaluated using both absorption- and phase-based imaging modalities, as well as visual inspection of the beam itself. Most problematic were the non-uniform intensities in some beams (Fig. 2*a* ▶). At its worst, this non-uniformity made imaging impractical. Fortunately, in most cases, the non-uniformity occurred mostly around the edges and still allowed a suitably large region for imaging. Absorption imaging tests were conducted for both projection and CT imaging. Flat-dark-corrected images were devoid of artefacts, despite a visible line of lower intensity due to another competing reflection diffracting away intensity, *i.e.* a glitch in the beam (Fig. 2*b* ▶). In an effort to locate a region of the diffracted beam devoid of glitches, the Bragg angle was adjusted through a small range (∼2°). While this did not remove the glitches as desired, a pleasing result was the production of an extremely large and uniform beam, covering a region approximately 40 mm vertical (V) × 94 mm horizontal (H) diffracted from a white beam with an incident height of 6.5 mm (Fig. 2*c* ▶).

**Figure 3:**
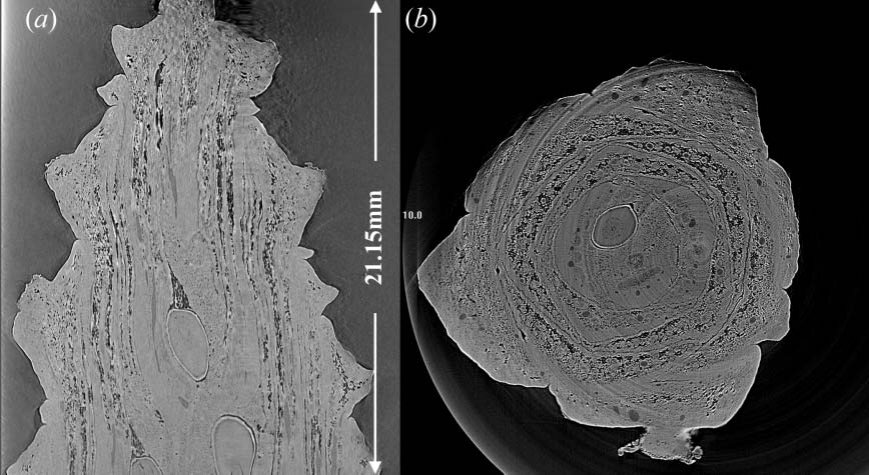
Micro-CT image of a pine cone. The image was captured in a single rotation. View (*a*) is an axial slice, view (*b*) is a sagittal slice. The vertical field of view of 21.15 mm would require seven rotations to capture without beam expansion.

The micro-CT imaging tests used a beam measuring 28 mm vertical × 62 mm horizontal. This beam was capable of completely filling the high-resolution (8.75 µm) Hamamatsu detector [AA-60 beam monitor coupled to C9300-124 CCD camera resulting in a field of view of 31.08 mm (H) × 23.31 mm (V)] regularly used for micro-CT. This expansion would allow objects up to about 21 mm in height (Fig. 3 ▶) to be imaged in a single rotation, rather than the vertical scanning method traditionally used at BMIT. This improvement would reduce scan times by as much as 85%.

**Figure 4:**
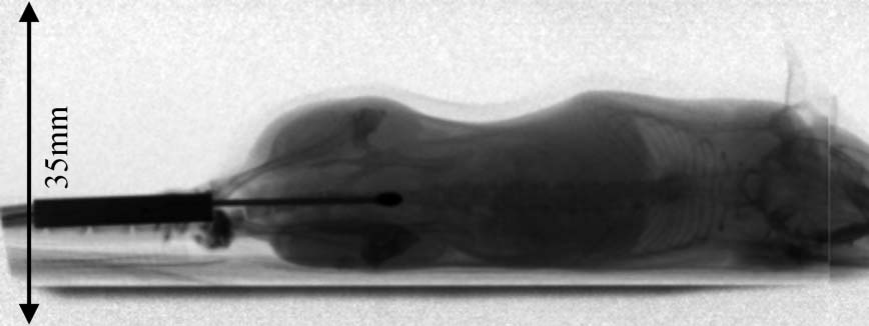
Flat-dark-corrected frame from a movie of a live mouse captured with a 200 µm flat-panel detector (Hamamatsu C9252DK-14) at 30 frames s^−1^. The movie is available online in the supporting information. [Supporting information for this paper is available from the IUCr electronic archives (reference: MO5075).] The vertical line on the right is an artefact of the detector, not the beam.

The 40 mm beam was used to capture live animal dynamic images using the flat-panel detector running at 30 frames s^−1^. This set-up allowed an entire adult mouse to be imaged laterally in a single shot (Fig. 4 ▶). Positioning the mouse vertically, this beam would be more than large enough to capture the entire lung region, allowing for dynamic lung imaging similar to the work reported by Lewis *et al.* (2005 ▶). All animal work was carried out in accordance with the Guidelines of the Canadian Council on Animal Care under the authority of the University (of Saskatchewan) Committee on Animal Care and Supply.

Flux was measured at 20.0 keV. The ion chamber measured a current of 316 pA at a ring current of 209.7 mA. The exposed area was 3.8 mm (V) × 17.4 mm (H) and the ion chamber path length was 15.1 cm. To protect the flat-panel detector, 6.66 mm of aluminium was used as a filter. Using the attenuation coefficients provided by NIST (Hubbell & Seltzer, 2004 ▶), the actual flux was calculated to be 2.5 × 10^4^ photons s^−1^ mm^−2^ mA^−1^, which would increase to 1.2 × 10^7^ photons s^−1^ mm^−2^ mA^−1^ without the filter. This would produce a surface dose of 4 µGy s^−1^ mA^−1^ with the filter and 2 mGy s^−1^ mA^−1^ without. Using this technique and the beamline’s Bragg double-crystal monochromator at 20 keV, the flux was measured to be 1.2 × 10^4^ photons s^−1^ mm^−2^ mA^−1^, which would increase to 5.7 × 10^6^ photons s^−1^ mm^−2^ mA^−1^ without the filter.

## 4. Discussion   

During all attempts at beam expansion, it was not found possible to create a perfectly uniform beam such as that produced by the beamline’s flat Bragg double-crystal monochromator. The diminished expansion (2.0×) during the 3× attempt may be explained by this non-uniformity as the image taken of the diffracted beam may have overlapped a region of low intensity. While the (1,1,1) wafers did appear to be free of glitches, the beam they produced lacked the uniformity required for high-quality imaging. It is suspected that the four bar bending system produces imperfect cylindrical bending due to elasticity in the bending bars and wafers, variations in crystal thickness, anticlastic bending of the crystals and non-parallel bending bars. This creates distortion in the crystals that prevents them from matching perfectly throughout the entire beam region, regardless of relative angle or distance. In future work the aim is to design a rigid bender with fixed bending radii so that the crystal will be forced into place.

The rigid frame bender will also provide an excellent heat sink for cooling the crystal with a liquid-metal (*i.e.* In/Ga) interface between the frame and the silicon. For these experiments, the maximum heat-load on the first crystal was calculated to be less than 25 W. During regular imaging, the filters used to protect the detector reduced the heat load to under 200 mW.

**Figure 5:**
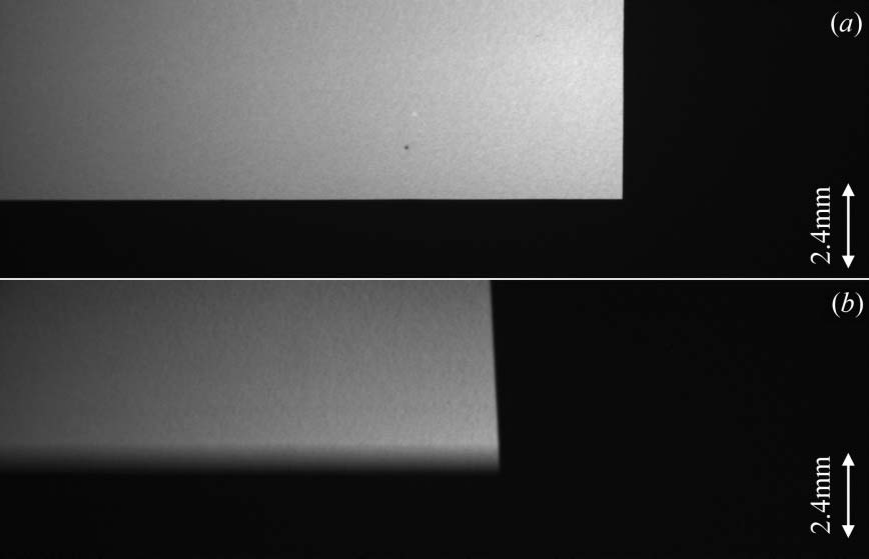
Vertical and horizontal knife-edge placed at (*a*) 140 mm and (*b*) 5135 mm sample–detector distance.

A knife-edge placed horizontally in the expanded beam revealed significant vertical blurring which increased with the distance between the edge and detector (Fig. 5 ▶). The blurring was not present in the horizontal direction, as a knife-edge placed vertically produced a sharp image at all distances. These results indicate that the X-rays exiting the second crystal are parallel horizontally but not vertically. The vertical beam divergence can be explained by diffraction occurring in-depth within both crystals producing a polychromatic focus and allowing rays to exit the same point in the second crystal but at different angles. This ‘Borrmann fan’ effect is known to occur in the Laue crystal during the process of dynamical diffraction. This effect increases the beam size and apparent source size in the diffraction plan and reduces the coherence of the beam. Such a ‘divergence effect’, if not controlled, will destroy the possibility of phase contrast in the vertical direction.

## 5. Conclusion   

A proof-of-principle study was carried out to determine whether a bent Laue beam expander could be developed for biomedical imaging applications. Beam expansion was successfully performed under a variety of conditions with expansions ranging from 2× to 7.7×. The measured flux per unit area was comparable with that available with the flat Bragg double-crystal monochromator currently used in the beamline. The increase in total photon count while expanding the beam size is made possible by the enhanced bandwidth of the bent Laue double-crystal monochromator. Some initial experiments were performed to demonstrate the viability and usefulness of the method. Problems that were identified include beam divergence after the second crystal as well as non-uniformity of the beam. The latter problem will be addressed by better control over the crystal and bending process but the beam divergence effect will require further study of ways to minimize or eliminate this phenomenon.

## Supplementary Material

Movie of live animal dynamic imaging, referenced in the text.. DOI: 10.1107/S1600577514003014/mo5075sup1.avi

## Figures and Tables

**Table 1:** Summary of expansion results and energy parameters

Attempt	Incident height (mm)	Diffracted height (mm)	Expansion factor	Silicon wafer	Reflection type	Bragg angle	Energy (keV)
Proof-of-principle	2.5	9.0	3.6	(1,1,1)	(1,1,1)	3.42°	33.16
Target of 3×	2.1	4.2	2.0	(5,1,1)	(2,2,0)	6.56°	28.3
Target of 5×	2.9	15.0	5.2	(5,1,1)	(2,2,0)	6.56°	28.3
Target of 7×	3.0	23.0	7.7	(5,1,1)	(2,2,0)	6.56°	28.3
Micro-CT imaging	4.0	28.0	7.0	(1,1,1)	(1,1,1)	6.56°	17.3
Dynamic imaging	6.5	40	6.2	(1,1,1)	(1,1,1)	6.31°	18.0
Flux	0.54	3.8	7.0	(1,1,1)	(1,1,1)	5.67°	20.0
